# The burden of COVID-19 based on disability-adjusted life years: a systematic review of available evidence

**DOI:** 10.3389/fpubh.2025.1401726

**Published:** 2025-02-24

**Authors:** Eshagh Barfar, Behzad Raei, Salman Daneshi, Fatemeh Bagher Barahouei, Kiavash Hushmandi

**Affiliations:** ^1^Health Promotion Research Center, Zahedan University of Medical Sciences, Zahedan, Iran; ^2^Department of Health, Safety, and Environment Management, School of Public Health, Zanjan University of Medical Sciences, Zanjan, Iran; ^3^Department of Public Health, School of Health, Jiroft University of Medical Sciences, Jiroft, Iran; ^4^M.Sc. of Health Care Management, Health Technology Assessment Center, Mashhad University of Medical Sciences, Mashhad, Iran; ^5^Nephrology and Urology Research Center, Clinical Sciences Institute, Baqiyatallah University of Medical Sciences, Tehran, Iran

**Keywords:** burden, COVID-19, disability-adjusted life years, DALYs, YLL, years of life lost, YLD, years lived with a disability

## Abstract

**Background:**

The present study tries to evaluate and summarize the available evidence to provide insights into the COVID-19 burden worldwide using disability-adjusted life years (DALYs) and compare the level of damage across countries during this pandemic.

**Method:**

We conducted a systematic review following the preferred reporting items for systematic reviews and meta-analyses (PRISMA) guidelines to investigate the global burden of COVID-19. Studies were identified through searches conducted on Ovid Medline, Cochrane, Science Direct, Scopus, and PubMed databases as well as, the Google Scholar search engine. All stages of the search, study selection, qualitative assessment, and data extraction were carried out by two authors separately. Any disagreement among reviewers was resolved by discussion.

**Results:**

The total DALYs incurred by COVID-19 varied widely among nations, with rates per 100,000 population ranging from approximately 5 in Korea to 5,363 in the US. Deaths due to COVID-19 could substantially impact years of life lost (YLLs), emerging as a major contributing factor to DALYs. Furthermore, unlike in high-income countries, a significant proportion of YLLs in low- and middle-income countries is associated with individuals dying at younger ages. Years lived with disability (YLDs) were also identified as a minor contributing factor to DALY estimates associated with COVID-19.

**Conclusion:**

Our findings from this investigation provide valuable insights into the impacts of COVID-19 on global health that may be an important basis for assessing its global burden, facilitating international comparisons, and allocating efforts to manage the epidemic. However, challenges persist in identifying and quantifying the economic costs and non-health effects of the event on an international scale.

## Background

In late 2019, cases of pneumonia of unknown origin were reported among some patients in China. The rapid spread of the disease led to a nationwide epidemic, followed by an international public health crisis. By early 2020, the World Health Organization (WHO) named it COVID-19, which stands for coronavirus disease 2019 (1). The disease is a severe acute respiratory syndrome that causes various symptoms and disabilities in patients (2). In a short period, COVID-19 faced the people of the world with various health, socio-economic, and political problems (3).

Following the rapid spread of this virus and the increase in infected and deceased patients, WHO declared it a pandemic in March 2020 (4). This disease has many similarities with the severe acute respiratory syndrome (SARS) and Middle East respiratory syndrome (MERS), but there are also obvious differences between them. The disease death rate is 2.3%, slightly lower than SARS (9.5%) and much lower than MERS (34.4%) (5). Also, COVID-19 has a high transmission power compared to SARS and MERS. It has unique characteristics that make it much more difficult to control and treat than previous coronaviruses (6).

Statistically, the risk of acquiring COVID-19 increases with older age. The death rate for people under 39 years old is about 2%, and this rate increases with age (4). The results of surveys have shown that the COVID-19 virus was more dangerous for men (with a death rate of 2.8%) than for women (with a death rate of 1.7%) (5). Additionally, 81% of the cases are mild, and a small percentage of patients show acute symptoms of the disease (6).

The effects of COVID-19 in different countries are different in incidence and mortality (7). Numerous efforts have been made to understand the impact of COVID-19 on health using mortality-based measures (8, 9), intensifying the need to account for years of life lost particularly (10). Understanding and quantifying the impact of the combination of disease morbidity and mortality is a fundamental step in standardizing the comparison between countries and quantifying the impact of COVID-19 compared to other causes of disease and injuries (11). Estimating summary measures of population health, such as potential disability-adjusted life years (DALYs), can achieve this. The DALYs related to COVID-19 are calculated as the sum of the years of life lost (YLLs) due to premature death and years lived with disability (YLDs) resulting from the disease. This aforementioned index is the most quantitative indicator that helps to determine health-related problems such as disease, death, and recovery. The basis of this feature and reputation lies in the fact that the index converts diverse and heterogeneous health problems into a single unit, namely lost time (12).

Therefore, the study of the burden of diseases provides a framework for determining priorities, assessing the effectiveness of investments, quantifying various dimensions of social development, and determining intervention strategies for researchers, policymakers, and community managers. By using the results of disease burden studies, the health manager derives the necessary research priorities, establishes the management priorities of their support department, specifies the priorities of health interventions, and assesses the possibility of determining the most efficient interventions (13). Considering the varying impacts of COVID-19 at the global level, as well as the importance of this evidence for healthcare providers and policymakers, and the necessity of optimal allocation of resources concerning this disease, the present study tries to evaluate and summarize the available evidence. The results of our study provide insights into the burden of COVID-19 worldwide and compare the level of damage across countries during this pandemic. This study is updating a systematic review study reported in 2023 (14).

## Methods

### Study design

This systematic review was conducted based on the preferred reporting items for systematic reviews and meta-analyses (PRISMA) guidelines. A systematic review method was selected to permit a robust and reproducible approach to structure a critical synthesis of the existing and current evidence. The study was approved by the Jiroft University of Medical Sciences (JMU) Ethics Committee (code: IR.JMU.REC.1400.036).

### Search strategy and data sources

To identify relevant papers published in academic journals in order to obtain data to investigate the global burden of disease, several databases were searched, including Ovid Medline, Cochrane, Science Direct, Scopus, PubMed, and finally, the Google Scholar search engine was also searched. Advanced and thematic search strategies were employed across these. The search was conducted on titles and abstracts. Available evidence on the burden of COVID-19 using the DALYs index was performed on December 25, 2024.

The combination of keywords and Medical Subject Headings (MeSH) was used: COVID-19, COVID-19, Coronavirus, Novel coronavirus, 2019-nCoV, Wuhan coronavirus, SARS-CoV-2, SARS2, nCov acute respiratory disease-2019, Coronavirus disease 2019, disability adjusted life year, DALY, year of life lost, YLL, year lost due to disability, and YLD. To combine terms, Boolean operators (AND, OR, and NOT) were employed. During this phase, a librarian was consulted to ensure the adequacy of the search strategy. In addition, the asterisk symbol (*) was used in search queries to enhance the comprehensiveness of the literature search. The search in each database was adapted appropriately. For example, the following search approach was implemented in the PubMed database: (((“COVID-19”[Title]) OR (coronavirus[Title])) OR (“SARS-CoV-2”[Title])) AND ((burden[Title]) OR (DALY*[Title])) OR (“disability adjusted life year*”[Title])) OR (“year* of life lost”[Title])) OR (YLL*[Title])) OR (“year* lost due to disability”[Title])) OR (“year* lived with disability”[Title])) OR (YLD*[Title])). We reviewed the references of selected papers to find additional studies not retrieved during the initial searches (reference by reference).

### Eligibility criteria

Our analyses included all English-language studies that reported evidence on the COVID-19 burden worldwide using the disability-adjusted life years (DALYs), years of life lost (YLLs), and years lived with disability (YLDs) indexes and were published in peer-reviewed journals between December 31, 2019, and December 25, 2024. Duplicate studies were removed after screening them based on their titles and abstracts.

### Study selection and data extraction

Two authors (S.D. and E.B.) separately performed the literature search and screened the studies, applying the inclusion and exclusion criteria based on the titles and abstracts. After initial screening, the full text of the studies was obtained and examined to ensure eligibility for developing the data extraction table. Data were extracted from all studies that were eligible and fulfilled the inclusion criteria for this review. The following data were extracted and analyzed: authors, publication date, country, study design, DALYs, YLLs, and YLDs.

### Quality assessment

For assessment of the completeness and quality of the included studies, we used the Critical Appraisal Skills Program (CASP) checklist for cross-sectional studies (www.casp-uk.net). This checklist employs a scoring system for quality assessment based on ten questions. Studies were rated as poor quality (0–4), medium quality (5–7), or high quality (8–10). Ultimately, the poor-quality studies were excluded from the current review.

### Synthesis of evidence

To express and synthesize the results of the included studies, we conducted a narrative synthesis of the overall evidence by comparing and contrasting the data. Data from the included studies was qualitatively described and presented. The authors met frequently to discuss and reach consensus on the findings.

## Results

### Electronic searches

**Figure 1:**
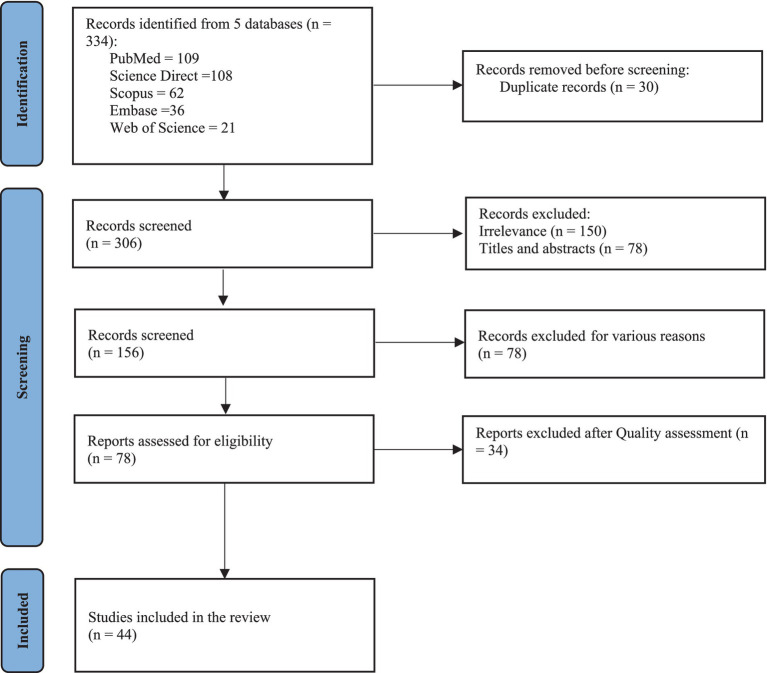
The PRISMA flowchart of the selection process for the studies.

An initial total of 336 studies were retrieved through a systematic search of published evidence from online databases. After reviewing titles and abstracts and removing duplicates, 156 studies were retained for review. The screening phase excluded 78 records, and an additional 34 studies were excluded based on eligibility assessment (Figure 1). The final included records were 44 studies. Of these, 34 focused on national or sub-national levels in various countries, with 19 conducted in Asia, 9 in Europe, and 7 in America. The remaining 9 studies provided international estimates produced by the Global Burden of Disease (GBD) project. While one research measured the burden to evaluate the effectiveness of alternative public health interventions in controlling the pandemic (1), most of the studies attempted to estimate the burden of COVID-19 to inform public health planning. The characteristics of the studies included in this systematic review are shown in Table 1.

**Table 1:** The general characteristics of the reviewed studies.

No.	Author	Duration time	Country (S)	Study design	Total DALYs (per population)	Total YLLs (per population)	Total YLDs (per population)	DALYs for males (per population)	YLLs for males (per population)	YLDs for males (per population)	DALYs for females (per population)	YLLs for females (per population)	YLDs for females (per population)
1.	Singh et al. (6)	11 months	India	Cross-sectional	14,100,422 (1,022 per 100,000 person)	13,992,709 (1,014 per 100,000 person)	105,784 (8 per 100,000 person)	N/A (1,270 per 100,000 person)	N/A (1,261 per 100,000 person)	N/A (10 per 100,000 person)	N/A (758 per 100,000 person)	N/A (752 per 100,000 person)	N/A (6 per 100,000 person)
2.	Oh et al. (10)	3 months	Global (30 countries)	Cross-sectional	Not reported	4,072,325 (1,593.72 per 100,000 person)	Not reported	Not reported	2,363,410 (104.68 per 100,000 person)	Not reported	Not reported	1,708,915 (77.78 per 100,000 person)	Not reported
3.	McDonald et al. (19)	10 months	Netherlands	Cross-sectional	273,500 (1,570 per 100,000 person)	271,859 (N/A)	1,641 (N/A)	Not reported	Not reported	Not reported	Not reported	Not reported	Not reported
4.	Vasishtha et al. (28)	12 months	India	Cross-sectional	N/A (6.12 per 1,000 person)	N/A (6.07 per 1,000 person)	N/A (0.06 per 1,000 person)	Not reported	Not reported	Not reported	Not reported	Not reported	Not reported
5.	Azarbakhshet al. (11)	4 months	Iran	Cross-sectional	Not reported	8,413 (2.77 per 1,000 person)	Not reported	Not reported	4,722 (3.06 per 1,000 person)	Not reported	Not reported	3,691 (2.46 per 1,000 person)	Not reported
6.	Mirzaei al. (15)	12 months	Iran	Cross-sectional	N/A (973 per 100,000 persons)	N/A (957 per 100,000 persons)	N/A (16 per 100,000 persons)	1,082 (per 100,000 persons)	Not reported	Not reported	861 (per 100,000 persons)	Not reported	Not reported
7.	Yousefi et al. (9)	15 months	Iran	Cross-sectional	Not reported	249,309 (957 per 100,000 person)	Not reported	Not reported	138,098 (18,761 per 10,000 person)	Not reported	Not reported	111,211 (16,385 per 10,000 person)	Not reported
8.	Taheri Soodejani et al. (16)	12 months	Iran	Cross-sectional	23,472 (N/A)	23,385 (N/A)	87 (N/A)	12,992 (22.2 per 1,000 person)	12,947 (N/A)	Not reported	10,480 (19 per 1,000 person)	10,438 (N/A)	Not reported
9.	Min-Woo et al. (7)	3 months	South Korea	Prospective, cohort of patient	2,531 (4.930 per 100,000 person)	2,270.7 (4.423 per 100,000 person)	260.3 (0.507 per 100,000 person)	Not reported	Not reported	Not reported	Not reported	Not reported	Not reported
10.	Nurchis et al. (5)	2 months	Italy	Observational	121,449 (2.01 per 1,000 person)	Not reported	Not reported	82,020 (N/A)	81,718 (N/A)	302 (N/A)	39,429 (N/A)	39,096 (N/A)	333 (N/A)
11.	Wang et al. (29)	12 months	USA	Cross-sectional	Not reported	496,998 (12.72 per person) in New York State	Not reported	Not reported	Not reported	Not reported	Not reported	Not reported	Not reported
						375,815 (15.13 per person) in New York City							
12	Silva et al. (30)	4 months	Brazil	Ecological	5,825.35 (N/A)	98.88% of DALYs	1.12% of DALYs	N/A (1,475.94 per 1,000 persons)	Not reported	Not reported	N/A (674.23 per 100,000 persons)	Not reported	Not reported
13.	Rommel et al. (13)	12 months	Germany	Cross-sectional	305,641 (368.2 per 100,000 person)	303,608 (N/A)	2,033 (N/A)	Not reported	Not reported	N/A (2.51 per 100,000 persons)	Not reported	Not reported	N/A (2.39 per 100,000 persons)
14.	John et al. (31)	17 months	India	Cohort-based observational	24,592.9 (709.2 per 1,000,000 person) in 2020	24,573.9 (N/A) in 2020	19.1 (N/A) in 2020	Not reported	Not reported	Not reported	Not reported	Not reported	Not reported
					140,481.3 (4,050.99 per 1,000,000 person) in 2021	139,327.5 (N/A) in 2021	1,153.7 (N/A) in 2021	Not reported	Not reported	Not reported	Not reported	Not reported	Not reported
15.	Fan et al. (17)	15 months	Global	Cross-sectional	31,930,000 (427.4 per 100,000 person)	96.22% of DALYs	13.78% of DALYs	Not reported	Not reported	Not reported	Not reported	Not reported	Not reported
16.	Salinas-Escudero et al. (18)	10 months	Mexico	Cross-sectional	2,165,424.5 (1,055 per 100,000 person)	2,126,222 (1,663.8 per 100,000 person)	39,202 (30.7 per 100,000 person)	Not reported	Not reported	Not reported	Not reported	Not reported	Not reported
17.	Cuschieri et al. (32)	12 months	Malta	Cross-sectional	5,478 (N/A)	5,229 (1,593.72 per 100,000 person)	157 (N/A)	Not reported	Not reported	Not reported	Not reported	Not reported	Not reported
18.	Pifarré Arolas et al. (4)	9 months	81 countries	Observational	Not reported	20,507,518 (N/A)	Not reported	Not reported	Not reported	Not reported	Not reported	Not reported	Not reported
19.	Gianino et al. (12)	10 months	16 European countries	Observational	852,790 (4,354 per 100,000 person)	835,685 (N/A)	17,105 (N/A)	Not reported	Not reported	Not reported	Not reported	Not reported	Not reported
20.	Zhao et al. (1)	2 months	China	Observational	38,348 (N/A)	32,575 (N/A)	822 (N/A)	Not reported	Not reported	Not reported	Not reported	Not reported	Not reported
21.	Quast et al. (33)	13 months	USA	Cross-sectional	Not reported	9,655,279 (297.5 per 10,000 person)	Not reported	Not reported	Not reported	Not reported	Not reported	Not reported	Not reported
22.	Ugarte et al. (34)	8 months	17 countries	Observational	Not reported	4,210,654 (N/A)	Not reported	Not reported	Not reported	Not reported	Not reported	Not reported	Not reported
23.	Moran et al. (8)	12 months	Republic of Ireland	Observational	51,532.1 (N/A)	50,858 (N/A)	674.2 (N/A)	Not reported	Not reported	Not reported	Not reported	Not reported	Not reported
24.	Lozano et al. (35)	12 months	Colombia	Observational	49,243 (2,692 per 100,000 person)	49,131 (2,686 per 100,000 person)	111.9 (6.1 per 100,000 person)	N/A (3,353.97 per 100,000 person)	N/A (3,348.14 per 100,000 person)	N/A (5.83 per 100,000 person)	N/A (2,037.82 per 100,000 person)	N/A (2,031.42 per 100,000 person)	N/A (6.40 per 100,000 person)
25.	Chen et al. (21)	24 months	USA	Observational	15,300,000 (5,363 per 100,000 person)	14,430,000 (5,148 per 100,000 person)	600,000 (215 per 100,000 person)	Not reported	Not reported	Not reported	Not reported	Not reported	Not reported
26.	Tsai et al. (36)	22 months	Taiwan	Observational	1,004.13 (N/A)	998.74 (99.5% of DALYs)	5.39 (N/A)	N/A (641.58 per 100,000 person)	Not reported	Not reported	N/A (361.95 per 100,000 person)	Not reported	Not reported
27.	Tan et al. (37)	2 months	China	Observational	47,646 (N/A) for the Real-world strategy	20,063 (N/A) for the Real-world strategy	27,583 (N/A) for the Real-world strategy	Not reported	Not reported	Not reported	Not reported	Not reported	Not reported
					569,715 (N/A) for the Routine strategy	234,934 (N/A) for the Routine strategy	334,781 (N/A) for the Routine strategy	Not reported	Not reported	Not reported	Not reported	Not reported	Not reported
					21 (N/A) for the Stricter strategy	0 (N/A) for the Stricter strategy	21 (N/A) for the Stricter strategy	Not reported	Not reported	Not reported	Not reported	Not reported	Not reported
28.	Swain et al. (38)	24 months	India	Cross-sectional	1,924,107 (N/A) in 2020	Not reported	Not reported	1,315,054 (1.90 per 1,000 person) in 2020	Not reported	Not reported	609,053 (0.93 per 1,000 person) in 2020	Not reported	Not reported
					4,340,726 (N/A) in 2021	Not reported	Not reported	2,965,184 (4.23 per 1,000 person) in 2021	Not reported	Not reported	1,375,342 (2.08 per 1,000 person) in 2021	Not reported	Not reported
					808,124 (N/A) in 2022	Not reported	Not reported	546,779 (0.77 per 1,000 person) in 2022	Not reported	Not reported	261,345 (0.39 per 1,000 person) in 2022	Not reported	Not reported
29.	Lundberg et al. (39)	17 months	Sweden	Observational	Not reported	75,151 (N/A)	Not reported	Not reported	43,384 (N/A)	Not reported	Not reported	31,768 (N/A)	Not reported
30.	Traebert et al. (40)	12 months	Brazil	Ecological	4,496.9 (883.8 per 100,000 person)	4,285.5 (842.2 per 100,000 person)	211.4 (41.5 per 100,000 person)	2,693.1 (1,098 per 100,000 person)	2,587 (1,054.8 per 100,000 person)	106.1 (43.3 per 100,000 person)	2,693.1 (1,098 per 100,000 person)	1,698.5 (644.4 per 100,000 person)	105.3 (39.9 per 100,000 person)
31.	Howe et al. (41)	4 months	Australia	Observational	50,900 (N/A)	Not reported	7,035 (N/A)	Not reported	Not reported	Not reported	Not reported	Not reported	Not reported
32.	John et al. (42)	24 months	India	Cohort-based observational	Not reported	Not reported	Not reported	190,568 (N/A)	Not reported	Not reported	117,310 (N/A)	Not reported	Not reported
33.	Gomes et al. (43)	12 months	Canada	Cross-sectional	Not reported	3,066,440 (12.5 per 1,000 person) in 2020	Not reported	Not reported	1,871,371 (15.4 per 1,000 person) in 2020	Not reported	Not reported	1,195,069 (9.6 per 1,000 person) in 2020	Not reported
					Not reported	5,512,380 (22.1 per 1,000 person) in 2021	Not reported	Not reported	3,198,167 (25.8 per 1,000 person) in 2021	Not reported	Not reported	2,314,213 (18.5 per 1,000 person) in 2021	Not reported
34.	Alinia et al. (44)	20 months	Iran	Observational	23,316.5 (1,385.3 per 100,000 person)	23,060.3 (N/A)	256.2 (N/A)	127,509 (1,494 per 100,000 person)	12,622.5 (N/A)	128.4 (N/A)	10,565 1,273.4 per 100,000 person)	10,437.8 (N/A)	127.8 (N/A)
35.	Wang et al. (29)	24 months	Canada	Observational	Not reported	Not reported	Not reported	N/A (6.493 per 1,000 persons)	N/A (5.897 per 1,000 persons)	N/A (0.596 per 1,000 persons)	N/A (5.316 per 1,000 persons)	N/A (4.654 per 1,000 persons)	N/A (0.662 per 1,000 persons)
36.	Espinosa et al. (45)	24 months	50 countries	Observational	Not reported	85,649,579 (398.9 per 10,000 persons)	Not reported	Not reported	N/A (3,000 per 10,000 persons)	Not reported	Not reported	N/A (2,000 per 10,000 persons)	Not reported
37.	Haneef et al. (46)	5 months	France	Observational	990,710 (1,472 per 100,000 person)	982,531 (1,460 per 100,000 person)	8,179 (13 per 100,000 person)	Not reported	559,784 (N/A)	1,824 (N/A)	Not reported	422,747 (N/A)	2,147 (N/A)
38.	Xie et al. (47)	27 months	USA	Cohort-based observational	N/A (287.43 per 100 persons)	Not reported	Not reported	Not reported	Not reported	Not reported	Not reported	Not reported	Not reported
39.	Devleesschauwer et al. (48)	22 months	Belgium	Observational	253,577 (2,206.4 per 100,000 person) in 2020	249,714 (2,172.8 per 100,000 person) in 2020	3,863 (33.6 per 100,000 person) in 2020	Not reported	Not reported	Not reported	Not reported	Not reported	Not reported
					139,281 (1,208.9 per 100,000 person) in 2021	130,979 (1,136.8 per 100,000 person) in 2021	8,303 (72.1 per 100,000 person) in 2021	Not reported	Not reported	Not reported	Not reported	Not reported	Not reported
40.	Shedrawy et al. (49)	19 months	Sweden	Observational	152,877 (1,419 per 100,000 person)	151,778 (N/A)	1,099 (N/A)	Not reported	Not reported	Not reported	Not reported	Not reported	Not reported
41.	Šantrić Milićević et al. (50)	22 months	Serbia	Observational	Not reported	Not reported	73 (N/A) in 2020	Not reported	Not reported	40.6 (5.1 per 100,000 person) in 2020	Not reported	Not reported	32.4 (3.6 per 100,000 person) in 2020
					Not reported	Not reported	130.3 (N/A) in 2021	Not reported	Not reported	65.1 (8.2 per 100,000 person) in 2021	Not reported	Not reported	65.2 (7.3 per 100,000 person) in 2021
42.	Bokaie et al. (20)	25 months	Iran	Observational	2,376,552 (2,860 per 100,000 person)	2,361,066 (2,842 per 100,000 person)	15,485.9 (18.6 per 100,000 person)	1,308,081 (3,061 per 100,000 person)	1,300,211 (1,530 per 100,000 person)	7,870 (13.6 per 100,000 person)	1,068,471 (2,573 per 100,000 person)	1,060,855.7 (1,248.5 per 100,000 person)	7,615.8 (13.7 per 100,000 person)
43.	Damiri et al. (51)	24 months	Iran	Cross-sectional	665,823 (1,603 per 100,000 person) in 2020	664,230 (1,599 per 100,000 person) in 2020	1,593 (3.8 per 100,000 person) in 2020	371,205 (1,738 per 100,000 person) in 2020	370,361 (1,734 per 100,000 person) in 2020	844 (3,95 per 100,000 person) in 2020	294,619 (1,459 per 100,000 person) in 2020	293,868 (1,456 per 100,000 person) in 2020	751 (3.7 per 100,000 person) in 2020
					928,393 (2,234 per 100,000 person) in 2021	925,457 (2,227 per 100,000 person) in 2021	2,936 (7.1 per 100,000 person) in 2021	485,490 (2,273 per 100,000 person) in 2021	484,046 (2,266 per 100,000 person) in 2021	1,444 (6.76 per 100,000 person) in 2021	442,792 (2,193 per 100,000 person) in 2021	441,262 (2,186 per 100,000 person) in 2021	1,530 (7.6 per 100,000 person) in 2021
44.	Ferrari et al. (22)	24 months	Global	Systematic review	123,000,000 (1,482.1 per 100,000 person) in 2020	118,000,000 (1,420.1 per 100,000 person) in 2020	4,950,000 (62 per 100,000 person) in 2020	78,500,000 (1,978.8 per 100,000 person) in 2020	76,600,000 (1,930.3 per 100,000 person) in 2020	1,920,000 (48.4 per 100,000 person) in 2020	44,800,000 (1,033.2 per 100,000 person) in 2020	41,800,000 (957.6 per 100,000 person) in 2020	3,030,000 (75.6 per 100,000 person) in 2020
					212,000,000 (2,500.8 per 100,000 person) in 2021	198,000,000 (2,324.5 per 100,000 person) in 2021	14,300,000 (176.4 per 100,000 person) in 2021	132,000,000 (3,247.9 per 100,000 person) in 2021	126,000,000 (3,111.8 per 100,000 person) in 2021	5,470,000 (136.1 per 100,000 person) in 2021	80,200,000 (1,822.6 per 100,000 person) in 2021	71,500,000 (1,606 per 100,000 person) in 2021	8,790,000 (216.6 per 100,000 person) in 2021

### Disease models and data sources

All studies comprising this review employed direct estimating models to assess the COVID-19 burden, encompassing disease-related morbidity and mortality. The main foundation for these models was observational studies, typically derived from vital registration systems and surveillance data. Such records and data from various countries are heavily relied upon by GBD to develop cause-of-death models. The included studies predominantly adopted a prevalence-based approach and did not incorporate duration parameters due to the nature of cross-sectional studies.

### Years of life lost (YLLs) per 1000/100,000 population by gender and age groups

A substantial and growing body of literature has examined YLLs due to COVID-19 across different countries. The rate for YLLs was reported differently among studies as per 1,000, 10,000, or 100,000 population, or per death. Furthermore, the magnitude of these rates varied significantly across countries. For example, YLLs per death ranged from 8.5 years in Italy (5) to 16 years at a global level (4), and up to 31.7 years in India (6). Generally, the YLLs rate is higher in males than in females. Across 81 countries, the male-to-female ratio of YLLs for COVID-19 ranges from near parity, as seen in Canada or Finland, to more than double, such as in Peru, or fourfold, as in Taiwan (4). One prominent example is the Korean study, which found that, in all age groups except for those aged 30–39 years, females exhibited lower YLL rates per 100,000 population compared to males (7). Another study also supported this occurrence by providing results comparing male and female YLL rates over the age groups except for the 45–64 age group (8). This likewise occurs in studies that reported YLLs per death. In Iran, for instance, one study estimated that the average YLLs rate associated with COVID-19 deaths was 923 per 10,000 males and 862 per 10,000 females. The YLLs per mortality due to COVID-19 were also approximately 18 (15). The distribution of YLL rates among different age groups in the extensive literature review reveals a consistent pattern. Overall, there appears to be evidence indicating that YLL rates per given population increase with aging in both genders, with the largest proportions of YLLs borne by the oldest group (60+ or 65+). According to one study, YLLs caused by COVID-19 in individuals aged 60 years and above accounted for almost three-quarters of the total YLLs in the population (10). Likewise, another study reported that the proportion of YLLs was highest in males aged 70–79 years and females aged 80 years and older (7). This trend holds even for YLL estimations in absolute terms. Two studies showed that the highest YLLs were in both genders in the age groups of 60–69 years and 60–64 years (4, 9). However, Pifarré et al. (4). found the opposite pattern in low- and middle-income countries, where a large proportion of the YLLs was attributable to individuals dying at age 55 or younger.

### Years lived with disability (YLDs) per 1000/100,000 population by gender and age groups

Generally, YLDs were identified as a minor contributor to DALYs. According to the studies, the proportionate contribution of YLDs to DALYs due to COVID-19 is 10.3% (7), 2.4% (1), 2% (4), and 1.3% (8). The male-to-female ratio of YLDs per 1000/100,000 population ranges from 0.68 in Korea (7), 1.05 in Germany (13), to 1.66 in India (6). Certain studies have revealed that the YLD rates are higher among males than females (7, 8). Results of the research conducted in Iran, however, indicated that both males and females experienced the same number of years of life with the same disability (16). YLDs per 100,000 population were reported as 8 years in India and 16 years in Iran (6, 15). Despite some discrepancy in YLD distribution throughout age groups in both genders, the evidence confirms that younger age groups experience larger YLDs compared to older ones. In Korea, the YLDs rate per 100,000 population was highest in people aged 20–29 years, followed by those aged ≥80 years, 50–59 years, and 60–69 years. One study in Ireland found that the proportion of YLDs was the highest in those aged 25–44 years in both genders (8).

### Disability-adjusted life years (DALYs) per 1000/100,000 population by gender and age groups

The total DALYs incurred by COVID-19 varied widely among nations. Two studies reported 14,106,060 DALYs in India and 31,930,000 DALYs globally (17). Similarly, Salinas-Escudero et al. discovered that COVID-19 caused 2,165,425 DALYs in the Mexican population (18). Estimates for Germany and the Netherlands stood at 305,641 and 273,500 years, respectively (13, 19). DALYs per 100,000 population varied significantly among countries, ranging from 5 in Korea (7) to 368 in Germany (13), 1,570 in the Netherlands (19), 2,860 in Iran (20), 5,363 in the US (21), and 2,501 globally (22). When standardized based on population size, gender-specific DALY estimates associated with COVID-19 from several studies indicate that males bear a higher burden than females across all age groups (7). The age distribution of DALYs suggests that the highest DALY rates were observed in the age group 71–80 in India, 70–79 in Iran, and 80–89 years old in Korea, Ireland, and Italy, as well as globally (10).

## Discussion

The COVID-19 pandemic has resulted in unprecedented disruptions to health systems, society, and the global economy. To better understand the pandemic’s enormous impact, our systematic review presents the available evidence on the burden of COVID-19 worldwide (20).

According to the results, deaths from COVID-19 could substantially impact YLLs, serving as a major contributing factor for DALYs attributed to this disease. Various studies indicated that YLLs account for 98.88, 96.22, and 99.5% of DALYs. YLLs per death vary from 8.5 years in Italy and 12.5 years in Belgium to 16 years globally and 31.7 years in India. Similar results have been confirmed by studies on other respiratory diseases (3, 23, 24). Additionally, in contrast to high-income countries, a significant proportion of YLLs due to COVID-19 in low- and middle-income countries is attributed to individuals dying at younger ages. While those succumbing to COVID-19 may belong to high-risk groups with lower life expectancies than the general population, the pandemic directly affects gross domestic product (GDP) at the macroeconomic level, primarily due to reduced productivity (3). Vaccination is a key strategy for reducing the disease burden and is regarded as a cost-effective public health intervention. According to a study in the United States, a 60% efficacy COVID-19 vaccine could prevent 31% of the expected deaths from the disease compared to no vaccine (23).

As demonstrated by the findings of the study, YLDs have been identified as a minor contributing factor to DALYs, with the reported relative contribution ranging from 1.3 to 10.3%. Additionally, YLDs were higher in the younger age groups when compared to the older ones. This suggests that although the number of deaths may be lower among younger groups, the sequelae and damage induced by the disease can severely affect their quality of life. It should be emphasized that disability weight plays a key role in estimating YLDs because it captures the magnitude of health loss associated with specific health consequences. The information gathered regarding COVID-19-related disability and sequelae is valuable. As YLD’s calculation allows for estimating the long-term consequences of the disease, it makes them an essential consideration for decision-making. The recovery time from the onset is approximately two weeks for moderate cases and three to six weeks for those with complicated cases. The long-term COVID-19 consequences, which have not yet been sufficiently covered in the research, could have a big influence on the disease burden assessment. For instance, the coronavirus family is noted to affect the central nervous system (25). Any type of future long-term neurological complication due to the virus will contribute to the increasing number of YLDs.

Based on our measurement, the total DALYs and DALYs per 1000/100,000 population associated with the direct health impact of COVID-19 vary widely across countries. Variations in the reported COVID-19 burden are expected due to factors such as age distribution, healthcare infrastructure, healthcare access, socioeconomic status, prevalence of comorbidities, and the duration of studies conducted in different countries. The extent of the disease burden could reflect the effectiveness of public health policies and societal commitment toward them. To halt virus transmission and mitigate the effects of YLDs and YLLs, effective management of COVID-19 necessitates well-coordinated strategies with strong local and international collaborations. This involves continued immunization strategies and possible social distancing policies whenever necessary.

The findings revealed a higher number of DALYs and YLLs due to COVID-19 among different age groups in males compared to females. Meanwhile, the number of YLDs attributable to COVID-19 in both genders varied across studies, yielding contradictory evidence. Several studies have reported that males are typically more vulnerable to severe diseases such as diabetes, cancer, and cardiovascular and liver diseases. Additionally, there is some evidence to support that females survive longer than males, even under extreme conditions like starvation (9, 26). Another likely explanation for the difference in COVID-19 mortality rates between males and females is differences in lifestyle factors, such as drinking alcohol and smoking consumption, which are more prevalent among males. Moreover, males are more likely to avoid using face masks compared to females (26).

The age pattern of YLLs and DALYs in different studies suggests that the highest YLLs and subsequent DALYs were detected in older age groups. This is mainly because of physiological modifications and comorbidity with other conditions, which in turn bring about a higher mortality rate among older COVID-19 patients. Furthermore, given that the older adult constitute a significant portion of the population in most countries that have measured the COVID-19 burden, these countries are severely affected by the epidemic, experiencing a high count of YLLs caused by this disease (27).

## Limitations

Our analysis has several limitations. Although we included international literature in our analysis, the majority of the research originated from nations where COVID-19 was known to be very prevalent. Thus, there is a need for more geographically diverse research. Additionally, significant heterogeneity in research design, population demographics, and reported measures made it difficult to do inter-study comparisons. This heterogeneity underscores the need for future research to adopt consistent definitions and measurement methodologies, enabling more reliable evidence synthesis. Moreover, reliance on observational data brings in possible biases, such as selection bias and confounding factors, which could impact the validity of the results. Finally, our analysis did not include studies that were published in languages other than English. This may restrict the comprehensiveness of our review, potentially skewing our understanding of COVID-19 burden in non-English-speaking regions.

## Conclusion

The reviewed studies illustrate that the virus can seriously affect human life, underscoring the importance of considering both health and socioeconomic factors when evaluating the pandemic’s effects. Our findings from this investigation provide valuable insights into the impacts of COVID-19 on global health that may be an important basis for assessing its global burden, facilitating international comparisons, and allocating efforts to manage the epidemic. However, challenges persist in identifying and quantifying the economic costs and non-health effects of the event on an international scale. An integrated approach can enhance our understanding of the pandemic’s actual effect and provide policymakers with a more holistic view, facilitating more informed decisions regarding intervention strategies. It calls for further research, particularly in underrepresented regions, that combines health impacts and economic evaluations to better capture the pandemic’s effects and improve public health responses.

## Data Availability

The original contributions presented in the study are included in the article/supplementary material, further inquiries can be directed to the corresponding author.
